# Process evaluation of an academic dissemination and implementation science capacity building program

**DOI:** 10.1017/cts.2023.630

**Published:** 2023-09-15

**Authors:** Clare Viglione, Borsika Rabin, Olivia Fang, Laura Sheckter, Gregory A. Aarons, Lauren Brookman-Frazee, Nicole A. Stadnick

**Affiliations:** 1 UC San Diego Altman Clinical and Translational Research Institute Dissemination and Implementation Science Center, University of California San Diego, La Jolla, CA, USA; 2 Herbert Wertheim School of Public Health and Human Longevity Science, University of California San Diego, La Jolla, CA, USA; 3 Department of Psychiatry, University of California San Diego, La Jolla, CA, USA; 4 Child and Adolescent Services Research Center, San Diego, CA, USA

**Keywords:** Dissemination and implementation science, capacity building, programs, evaluation, translational science

## Abstract

The UC San Diego Altman Clinical and Translational Research Institute Dissemination and Implementation Science Center (DISC) launched in 2020 to provide dissemination and implementation science (DIS) training, technical assistance, community engagement, and research advancement. DISC developed a program-wide logic model to inform a process evaluation of member engagement and impact related to DISC services. The DISC Logic Model (DLM) served as the framework for a process evaluation capturing quantitative and qualitative information about scientific activities, outputs, and outcomes. The evaluation involved a multimethod approach with surveys, attendance tracking, feedback forms, documentation of grant outcomes, and promotions metrics (e.g., Twitter engagement). There were 540 DISC Members at the end of year 2 of the DISC. Engagement in the DISC was high with nearly all members endorsing at least one scientific activity. Technical assistance offerings such as DISC Journal Club and consultation were most frequently used. The most common scientific outputs were grant submission (65, 39%), formal mentoring for career award (40, 24%), and paper submission (34, 21%). The DLM facilitated a comprehensive process evaluation of our center. Actionable steps include prioritizing technical assistance, strengthening networking opportunities, identifying streamlined approaches to facilitate DIS grant writing through writing workshops, as well as “office hours” or organized writing leagues.

## Introduction

Dissemination and implementation science (DIS) intends to bridge the gap between research, practice, and policy by building a knowledge base about how health information, effective interventions, and new clinical practices, guidelines, and policies are communicated and integrated for public health and health care service use in specific settings [1]. DIS research and training programs focused on capacity building have proliferated in recent years, including the National Institutes of Health Training Institute for Dissemination and Implementation Research in Cancer, Implementation Research Institute program, and the National Institute of Mental Health Division of Services and Intervention Research’s Dissemination and Implementation Research Program [2,3]. In this context, capacity building refers to the “process of individual and institutional development which leads to higher levels of skills and greater ability to perform useful research [4].” Examples of capacity building activities include targeted consultation, technical assistance for research teams, and the provision of educational materials and operational toolkits to guide researchers in the systematic application of DIS.

The goal of these activities is to strengthen DIS knowledge and skills of individuals, teams, health systems and organizations through various processes [5], as well as to advance the public health impact of research and practice through DIS. Although many DIS programs have similar training and capacity building goals, their methods, activities, operations, foci, and audiences may be different, leading to varied and nuanced capacity building activities and operations. Few programs have published evaluation reports. As such, evaluating the process and impact of these programs is essential to identify successes, challenges, and gaps in capacity building efforts.

Harmonized measures across programs could facilitate the identification of areas for opportunity, collaboration, and growth, but uptake of common measures have been limited [2]. Despite an increasing number of programs, there is a paucity of evaluation frameworks designed specifically for DIS capacity building programs. Existing frameworks (e.g., Brownson *et al*., Washington University Network of Dissemination and Implementation Researchers (WUNDIR) logic model; Cooke *et al*., Research Capacity Building Framework) have focused on academic outcomes (e.g., papers and presentations) [3,6]. For example, the WUNDIR program developed one of the first logic models with people, settings, and activities serving as pillars working synergistically to support capacity building [3]. WUNDIR connects scientific activities to different outputs for evaluation (e.g., DIS skills) [7].

In this paper, we outline a process evaluation of an academically housed DIS capacity building program, the UC San Diego Altman Clinical and Translational Research Institute Dissemination and Implementation Science Center (DISC). DISC launched in 2020 to advance DIS through training, technical assistance, community engagement, and research innovation. We describe the development of a capacity building model, the DISC Logic Model (DLM), to capture important academic dissemination products and scientific outcomes. We use the DLM to guide a process evaluation to (1) assess how engagement in DISC activities translates into scientific products, outputs, and outcomes and (2) explore how to improve the DISC using feedback from DISC members. To our knowledge, this is one of the first reports describing the evaluation of a DIS capacity building program.

## Methods

### Study Design

**Figure 1.**
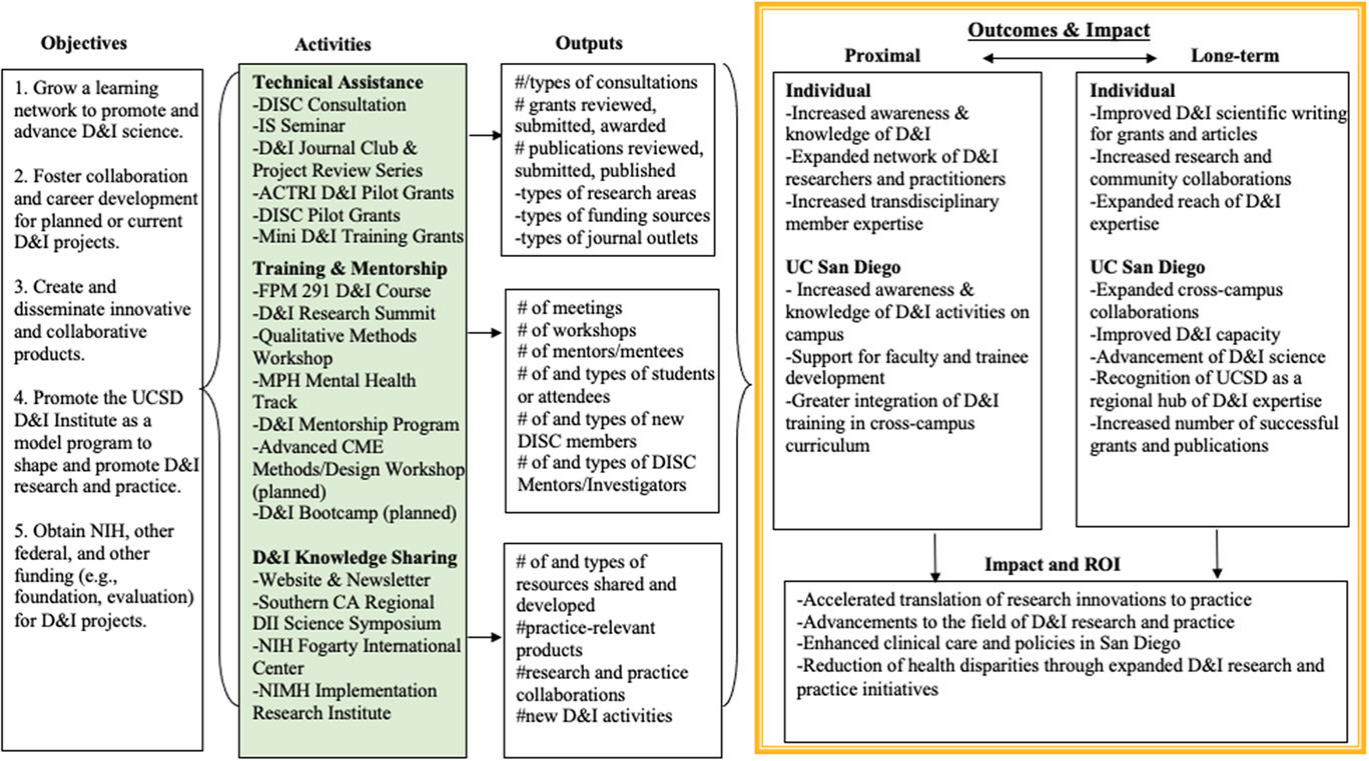
UC San Diego Dissemination and Implementation Science Center (DISC) logic model.

The DISC Evaluation for year 1 (2020) and year 2 (2021) was guided by the DLM (see Fig. 1) and includes a multimethod approach (e.g., surveys, attendance tracking, feedback forms, documentation of grant outcomes, etc.). The evaluation was designed by investigators and research staff from the DISC. Since data collected were anonymous program evaluation data, this protocol was not submitted for review.

### The UC San Diego Altman Clinical and Translational Research Institute Dissemination and Implementation Science Center

The DISC launched in January 2020 and provides (1) DIS trainings and workshops, (2) DIS technical assistance and resource sharing, (3) networking and community engagement, (4) pilot funding and grant review, and (5) expert DIS-specific consultation and mentoring. The DISC is led by the Executive Leadership team comprised of four faculty with expertise in DIS and independent, large-scale research programs in DIS with administrative support from a full-time Center Manager. The DISC was initially funded through a 3-year investment from UC San Diego Health Sciences to stimulate DIS research and practice at UC San Diego, its regional affiliates, and beyond. UC San Diego Health Sciences encompasses UC San Diego Health, the region’s only academic health system, UC San Diego School of Medicine, one of the nation’s top research-intensive medical schools, Skaggs School of Pharmacy and Pharmaceutical Sciences, Southern California’s first public school of pharmacy, and Herbert Wertheim School of Public Health and Human Longevity Science. DISC scientific and capacity building activities include the newsletters, events such as DISC implementation science seminars, DISC journal club, DISC workshops, DISC website, and consultation. DISC consultation services are provided by 12 DISC investigators from various departments (i.e., Public Health, Psychiatry, and Anthropology) who are experts in DIS. DISC Members who engage with DISC activities including consultations and workshops and contribute to DIS related grants, publications, and projects, are more likely to increase their capacity to perform DIS research and practice.

### DISC Member Participants

DISC Membership is free and available to the public. Individuals can register through the website (disc.ucsd.edu) on a rolling basis by completing the application form through Qualtrics. Applicants elect to be a DISC General Member or a DISC Investigator. General Members complete an annual survey and are eligible to receive pilot funding and individual consultation. Investigators can additionally request letters of support, promote achievements through DISC media (e.g., Twitter and newsletters), and receive priority consultation. Investigators are asked to contribute to two DISC services per year.

### Development of the DISC Logic Model (DLM)

DLM development occurred in three phases. The first phase consisted of building and adapting the logic model from the WUNDIR model and outlining activities inputs, short-term outcomes, and long-term outcomes relevant to the DISC. This process was initiated prior to the official launch, supported initial fundraising for the DISC, and was refined post-launch based on feedback from university leadership and key partners. The second phase consisted of piloting in year 1 (2020) which involved operationalizing each section of the model with concomitant activities such as DISC Consultation and DISC Journal Club and Works in Progress. The final phase included tailoring of the model to align with actual DISC activities and current resources and constraints after the end of year 1.

### DISC Evaluation Components

The DLM served as the framework for the multimethod DISC process evaluation examining scientific activities, outputs, and impact. Evaluation indicators presented align with DIS scientific activities (e.g., # and types of activities, attendance to events, etc.), DIS scientific outputs (e.g., DIS grant outcomes, DIS publications, other academic outcomes), and DIS outcomes (e.g., increased knowledge and awareness of DIS). The sources for the evaluation include the DISC Membership Application Form, DISC Annual Membership Evaluation Survey, DISC Consultation Request Form, DISC Consultation Outcomes Forms, Clockify Timetracker, and DISC Media Database (e.g., internal tracking of DISC events, #/types of dissemination products, and DISC media metrics). Evaluation forms are in Supplemental File 1.

### DISC Media and Communications

The DISC newsletters feature resources, events, job opportunities, member highlights, and recent publications. Newsletters are delivered via Mailchimp, a web application for e-newsletters which tracks analytics such as newsletters sent, newsletter open rate, and click rate. The DISC website (disc.ucsd.edu) is managed by UC San Diego and hosted through a centralized content system. Web analytics, tracked by SiteImprove.com, include visits, page views, unique and return visitors.

### DISC Membership Application Form

The 28-item DISC Membership Application form includes: institutional affiliation, level of DIS experience, keywords to describe expertise, event participation, as well as DIS-related needs. Results were analyzed by summarizing responses, displaying frequencies, and synthesizing themes.

### DISC Annual Evaluation Survey

The DLM served as the framework for a 37-item survey capturing quantitative and qualitative information about scientific activities, outputs, and impact. Items queried include activities and services participated in, activities supported through DISC membership, areas of improvement for DISC events, DISC website use, and types of resources that members would find of value. In year 1, the survey was distributed in February 2021, and in Year 2, the survey was distributed in February 2022. Surveys were sent to the membership listserv via Mailchimp with two reminders, one Twitter reminder and a $25 raffle incentive. Quantitative and qualitative data were descriptively summarized.

### DISC Consultation Evaluation System

Members receive free DISC consultation after completing an online request. An intake call (∼30 minutes) by the DISC Manager is conducted to inform matching of faculty DIS consultant(s) who lead an in-depth consultation meeting (∼1 hour). Subsequent meetings are scheduled based on needs of the consultee and consultant availability. REDCap forms evaluating satisfaction are distributed to consultees after 4–6 weeks and forms about long-term impact are distributed after 4-6 months. Consultants (*n* = 12) log hours for consultation via Clockify, a timekeeping program.

### DISC Qualitative Feedback

We ask for qualitative, open-ended feedback on our annual DISC membership form and in our workshop evaluation form. We ask respondents what they would like to see more of from the DISC, what they would like to change, and if they have feedback to guide DISC improvement. We developed themes after reviewing all open-ended feedback. One team member categorized responses by theme and the other team members reviewed and validated themes. Representative excerpts are featured.

## Results

### DISC Logic Model

The DLM appears in Fig. 1. Detailed description of DLM constructs is available in Supplemental File 2.

### DISC Inputs and Activities

#### Member characteristics

Tables 1 and 2 display DISC member characteristics aligning with DISC Inputs within the DLM. At the end of 2020 (year 1), there were 343 members including 279/343 (81%) DISC General Members and 64/343 (19%) DISC Investigators. At the end of 2021 (year 2), the member count had increased by 197, 157/197 (80%) DISC General Members and 40/197 (20%) DISC Investigators, to a new total of 540 members. The most common type of member was University Faculty (professors, research scientists) in year 1, 160/343 (47%) and year 2, 70/197 (36%). The proportion of student members (undergraduate and graduate) increased from year 1 (61/343 (18%) to year 2 (60/197 (30%)). Of total DISC Members (*n* = 540), most members identified as white (174/540, 32%) or Asian (64/540, 12%). Members reported multiple topics of interest: Psychiatry/Mental Health (280/540, 52%), Public Health (259/540, 48%), Health Services (217/540, 40%), Health Promotion (166/540, 31%), or Global Health (125/540, 23%). More than half reported novice or advanced beginner skills at time of registration in both year 1 (221/343, 64%) and year 2 (129/197, 66%). In terms of DIS experience, most members had not received funding for a DIS project (431/540, 81%) and had not published a DIS paper (380/540, 72%) at registration.

**Table 1.** DISC inputs and activities. Human resources

	Year 1 *N* (%)	Year 2 *N* (%)	Data source
*DISC Member Community^1^*			
*New DISC Members*	343	197	
*DISC General Members*	279 (81)	157 (80)	Membership Application Form
*DISC Investigators*	64 (19)	40 (20)	Membership Application Form
*Total DISC Members*	343	540^1^	
*DISC Member Characteristics^2^*			
*Professor (assistant, associate, etc.)*	160 (47)	70 (36)	Membership Application Form
*Research Scientist/Staff & Project Scientists*	55 (16)	31 (16)	Membership Application Form
*Clinician or Care Provider*	17 (5)	13 (7)	Membership Application Form
*Non Faculty Researchers*	15 (4)	9 (5)	Membership Application Form
*Post-Doctoral Candidates*	44 (13)	21 (11)	Membership Application Form
*Students (graduate, undergraduate, etc.)*	61 (18)	60 (30)	Membership Application Form
*Other*	16 (5)	16 (8)	Membership Application Form
*DISC Member Race/Ethnicity^3^*			
*White*	67 (20)	107 (54)	Membership Application Form
*Asian (Not Pacific Islander)*	22 (6)	42 (21)	Membership Application Form
*Latin American/Latino/Latinx*	19 (6)	32 (16)	Membership Application Form
*Black/African American (Not Hispanic)*	8 (2)	16 (8)	Membership Application Form
*Prefer to self-describe*	6 (2)	8 (4)	Membership Application Form
*Other Spanish/Spanish American*	3 (.9)	2 (1)	Membership Application Form
*Prefer not to answer*	3 (.9)	4 (2)	Membership Application Form
*American Indian/Alaskan Native*	3 (.9)	3 (2)	Membership Application Form
*Filipino/Pilipino*	2 (.5)	6 (3)	Membership Application Form
*Native Hawaiian/Other Pacific Islander*	1 (.3)	0 (0)	Membership Application Form
*Unknown*	225 (66)	4 (2)	Membership Application Form
*Topics of Research or Practice^4^*			
*Psychiatry/Mental Health*	195 (57)	85 (43)	Membership Application Form
*Public Health*	154 (45)	105 (53)	Membership Application Form
*Health Services*	132 (38)	85 (43)	Membership Application Form
*Health Promotion*	101 (29)	65 (33)	Membership Application Form
*Global Health*	75 (22)	50 (25)	Membership Application Form
*Health Policy*	70 (20)	59 (30)	Membership Application Form
*Aging*	58 (17)	13 (7)	Membership Application Form
*Pediatrics*	50 (15)	36 (18)	Membership Application Form
*Institutional Affiliation^5^*			
*University of California*	206 (60)	74 (38)	Membership Application Form
*Other Institution*	103 (30)	119 (61)	Membership Application Form
*California State University*	47 (14)	18 (9)	Membership Application Form
*County Health Department*	33 (10)	10 (5)	Membership Application Form
*VA San Diego*	30 (9)	8 (4)	Membership Application Form
*Rady Children’s Hospital San Diego*	25 (7)	5 (3)	Membership Application Form
*Level of DIS Skill^6^*			
*Novice*	124 (36)	65 (33)	Membership Application Form
*Advanced Beginner*	97 (28)	64 (33)	Membership Application Form
*Intermediate*	87 (25)	42 (21)	Membership Application Form
*Advanced*	35 (10)	25 (13)	Membership Application Form
*DIS Needs^7^*			
*Further training or mentoring in DIS*	208 (47)	133 (49)	Membership Application Form
*Help with DIS proposal*	117 (26)	79 (29)	Membership Application Form
*Help with DIS publication*	56 (13)	31 (11)	Membership Application Form
*Disseminating findings about a DIS product*	39 (9)	20 (7)	Membership Application Form
*Other*	24 (5)	10 (4)	Membership Application Form
*Prior DIS Grant Funding^8^*			
*Yes*	66 (20)	32 (16)	Membership Application Form
*No*	267 (80)	164 (84)	Membership Application Form
*Prior Publication of DIS Research^9^*			
*Yes*	88 (26)	60 (31)	Membership Application Form
*No*	246 (74)	134 (69)	Membership Application Form

DISC = UC San Diego Dissemination and Implementation Science Center; DIS = dissemination and implementation science.

1 Year 2 Total = Year 1 Total + Year 2 New Members.

2 Year 1 DISC Member Characteristics are calculated with the denominator = 343. Year 2 DISC Member Characteristics are calculated with the denominator = 197.

3 Year 1 DISC Race/Ethnicity data are calculated with the denominator = 343. Year 2 DISC Race/Ethnicity data are calculated with the denominator = 197.

4 Year 1 Types of Research and Practice are calculated with the denominator = 343. Year 2 Types of Research and Practice are calculated with the denominator = 197. Of note, respondents could select multiple options, so the proportions add up to more than 100%.

5 Year 1 Institutional Affiliations are calculated with the denominator = 343. Year 2 Institutional Affiliations are calculated with the denominator = 197. Of note, respondents could select multiple options, so the proportions add up to more than 100%.

6 Year 1 Levels of DIS Skill are calculated with the denominator = 343. Year 2 Levels of DIS Skill are calculated with the denominator = 197.

7 Year 1 DIS Needs are calculated with the denominator = 343. Year 2 DIS Needs are calculated with the denominator = 197. Of note, respondents could select multiple options, so the proportions add up to more than 100%.

8 Year 1 Prior Grant Funding are calculated with the denominator = 343. Year 2 Prior Grant Funding are calculated with the denominator = 197.

9 Year 1 Prior Publication of DIS Research are calculated with the denominator = 343. Year 2 Prior Publication of DIS Research are calculated with the denominator = 197.

**Table 2.** DISC inputs and activities. Scientific activities and outputs

	Year 1 *N* (%)	Year 2 *N* (%)	Data source
*Annual DISC Member Evaluation Respondents* ^1^	*165 (48)*	*101 (19)*	Annual Evaluation Form
DISC Activities^2^			
Reported participation in at least 1 Scientific Activity	164 (99)	101 (100)	Annual Evaluation Form
Reported participation in 3 or more Scientific Activities	130 (79)	67 (66)	Annual Evaluation Form
Reported participation in 5 or more Scientific Activities	64 (39)	43 (43)	Annual Evaluation Form
DIS Scientific Outputs^3^			
Grant submission	65 (39)	17 (17)	Annual Evaluation Form
Received formal mentoring for career award	40 (24)	5 (5)	Annual Evaluation Form
Paper submission	34 (21)	11 (11)	Annual Evaluation Form
Developed new scientific collaborator(s)	17 (17)	12 (12)	Annual Evaluation Form
Formed community partnership(s)	24 (15)	10 (10)	Annual Evaluation Form
Funded grant	24 (15)	5 (5)	Annual Evaluation Form
Published paper	17 (10)	5 (5)	Annual Evaluation Form
Scientific or Community Conference Presentation	13 (8)	0 (0)	Annual Evaluation Form
Operationalized new program or refined existing program	11 (7)	9 (9)	Annual Evaluation Form
Other	17 (10)	15 (15)	Annual Evaluation Form
DIS Grant Submission^4^			
Public Agency	47 (75)	12 (86)	Annual Evaluation Form
Private/Foundation	13 (21)	0 (0)	Annual Evaluation Form
Internal/Institutional Funds	3 (5)	2 (14)	Annual Evaluation Form
Most frequently visited DISC Website Pages^5^			
News & Events	67 (64)	44 (65)	Annual Evaluation Form
Tools & Resources	58 (55)	49 (72)	Annual Evaluation Form
Research Advancement	20 (19)	14 (21)	Annual Evaluation Form
Training & Education	53 (51)	38 (56)	Annual Evaluation Form
Institutional Equity			
*DISC is actively promoting equity* *(5=Strongly agree, 1=Strongly disagree)*	4.31 (0.8)	4.51 (0.7)	Annual Evaluation Form
General Satisfaction with DISC			
*Will you recommend DISC?* *(5=Extremely likely, 1=Extremely unlikely)*	4.34 (0.9)	4.45 (0.8)	Annual Evaluation Form
DIS Resources and Tools			
*DISC Newsletters* ^6^			
*Total Newsletters sent*	7	12	MailChimp Reports
*Newsletter open rate N (%)*	129 (38)	180 (33)	MailChimp Reports
*Newsletter click rate N (%)*	32 (9)	34 (7)	MailChimp Reports
*DISC Website*			
*Visits*	4,156	5,561	SiteImprov Reports
*Page Views*	11,436	15,527	SiteImprov Reports
*Unique visitors*	2,599	3,384	SiteImprov Reports
*Returning visitors*	181	147	SiteImprov Reports
*DISC Twitter*			
*Total Followers*	832	1,146	DISC Twitter Analytics
*Total Tweet Impression* ^7^	104,713	251,200	DISC Twitter Analytics
*New Followers*	592	314	DISC Twitter Analytics
DIS Training and Education			
DISC Journal Club			
*# Events*	12	12	Journal Club Sign-in Sheet
*Attendance M (SD)*	18.2 (5.7)	25.2 (7.2)	Journal Club Sign-in Sheet
*Satisfaction M (SD)* ^8^ *(1*–*10 rating)*	–	9.0 (0.6)	Journal Club Evaluation Form
DISC Implementation Science Seminars			
*# Events*	1	3	Seminar Sign-in Sheet
*Attendance M (SD)*	37 (0)	14.8 (3.5)	Seminar Sign-in Sheet
*Satisfaction (Y/N)*	100%	100%	Seminar Evaluation Form
DISC Workshop			
*# Events*	1	2	Workshop Sign-in Sheet
*Attendance M (SD)*	221 (0)	78.5 (33.2)	Workshop Sign-in Sheet
*Satisfaction M (SD), (1*–*5 rating)*	4.6 (0.8)	4.4 (0.2)	Workshop Evaluation Form

DISC = UC San Diego Dissemination and Implementation Science Center; DIS = dissemination and implementation science.

1 Year 1 Evaluation Respondents are calculated with the denominator of Total Members = 343. Year 2 Evaluation Respondents are calculated with the denominator of total members = 540.

2 DISC offered Scientific Activities include Monthly DISC Journal Club and Resources on DISC Website. The denominator is the number of survey respondents for the year (year 1 = 165 and Year 2 = 101). Of note, respondents could select multiple options, so the proportions add up to more than 100%.

3 The denominator is the number of survey respondents for the year (year 1 = 165 and year 2 = 101). Respondents could select multiple options, so the proportions add up to more than 100%.

4 The denominator is the number of respondents to the specific question about grant submissions (year 1 = 63 and year 2 = 14).

5 For and most frequently visited web pages, the denominator is the number of respondents to that specific question (year 1 = 105 and year 2 = 68).

6 open rate N: Number of times newsletters were opened. Open rate %: Percentage of newsletters opened by subscribers. Click rate N: Number of times any tracked link in newsletters are clicked. Click rate %: Percentage of newsletters that registered at least 1 click.

7 Total Tweet Impression: Times that a user is served a tweet in timeline or search results.

8 Satisfaction measurements: Journal Club Satisfaction Year 1: Did not start measuring satisfaction until year 2, Journal Club-Overall, how valuable was today’s DISC Journal Club? (1 Not Valuable at all– 10 Extremely Valuable), ISS-Would you recommend this program to others? (Y/N), Workshop-Overall I was satisfied with the workshop (1 Strongly Disagree – 5 Strongly Agree).

#### DISC annual evaluation

DISC Annual Evaluation results are in Tables 1 and 2. More than 95% of DISC Member respondents endorsed participation in at least one DISC Scientific Activity (e.g., workshops, consultations, seminars, etc.). There were 165 DISC member respondents to the Annual Evaluation in year 1 and 101 DISC member respondents to the Annual Evaluation in year 2. In year 1, the most common DIS Scientific Outputs were: grant submission (65/165, 39%), formal mentoring for career award (40/165, 24%), and paper submission (34/165, 21%). In year 2, the most frequent responses were grant submission (17/101, 17%), new scientific collaborator(s) (12/101, 12%), and other (15/101, 15%). Examples of “other” include consulting opportunities and presenting at DIS events.

#### DISC scientific and capacity building activities

The following evaluation items align with DLM Scientific Activities (e.g., # and types of activities, attendance to events, etc.). Year 1, the DISC distributed 7 newsletters with an average open rate of 45% and average click rate of 10% (Tables 1 and 2). In year 2, the DISC distributed 12 newsletters with an open rate of 19% and click rate of 8%. From year 1 to 2, visits increased from 4,156 to 5,561 and page views increased from 11,436 to 15,527. Unique visitors increased from 2,599 to 3,384. Returning visitors decreased from 181 to 147. At the end of year 1, the DISC Twitter had 832 followers, and 1,146 followers by the end of Year 2. Average DISC Journal Club attendance increased from 18 to 25. The DISC held one Implementation Science Seminar (ISS) in Year 1 with an attendance of 37. In Year 2, three ISS events were held with an average attendance of 15.

### DISC Outputs and Outcomes

The following evaluation items align with the DLM Outputs (e.g., # and types of consultations, # and types of activities, attendance to events, etc.) and DLM Outcomes (e.g., short-term outcomes of increased awareness and knowledge of DIS and long-term outcome of improved DIS capacity). The DISC completed 68 consultations in Year 1 and 70 consultations in Year 2 (Table 3). Most consultations in Year 1 were for grant support 48/68 (71%), project implementation 11/68 (16%), and networking/career development 7/68 (10%). In Year 2, 37/70 (53%) consultations were for grant support and 21/70 (30%) were for project implementation. 43/138 (31%) proposal consultations were for K or R-level NIH grants. Most consultees (41/42) agreed that consultations were “very valuable.” Consultees “strongly agreed” that consultations were useful, the consultant actively listened to questions, and that they would recommend DISC consultation to colleagues (4.9 out of 5 average across Year 1 and 2). Consultees also reported that needs were addressed during the consultation and connections and/or collaborations were shared afterwards (4.7 out of 5 average across Years 1 and 2). With respect to impact, from available data, 13/68 (19%) grants that had DISC consultation support were funded and 8/68 (12%) were not funded in Year 1. In Year 2, 11/68 (16%) grants with DISC consultation support were funded, while 13/68 (19%) grants are still pending or under review. Based on available Clockify data, consultation hours per project decreased from 9.8 hours in Year 1–6.4 hours in Year 2. In terms of satisfaction, those who attended ISS events in Year 1 and 2 responded “Yes” to the question, “Would you recommend this program to others?.” Year 1 included one workshop with an attendance of 221 and average satisfaction rating of 4.6 out of 5. Year 2 included two workshops with an average attendance of 79 and average satisfaction of 4.4 out of 5.

**Table 3.** Consultation outputs and outcomes

	Year 1 *N* (%)	Year 2 *N* (%)	Total	Source
DIS Consultation-Scientific Outputs				
*DISC Consultations*				
*Completed Consultations*	68 (100)	70 (100)	138 (100)	Consultee Service Request Forms
*DISC Consultation Types^1^*				
*Grant Support*	48 (71)	37 (53)	85 (62)	Master Tracker
*Publication Support*	2 (3)	4 (6)	6 (4)	Master Tracker
*Project Implementation*	11 (16)	21 (30)	32 (23)	Master Tracker
*Networking / Career Development*	7 (10)	6 (9)	13 (9)	Master Tracker
*Other*	0 (0)	2 (3)	2 (1)	Master Tracker
*Types of Grants*				
*NIH R*	15 (31)	16 (43)	31 (22)	Consultee Service Request
*NIH K*	5 (10)	7 (19)	12 (9)	Consultee Service Request
*NIH P*	1 (2)	2 (5)	3 (2)	Consultee Service Request
*NIH UG*	2 (4)	0 (0)	2 (1)	Consultee Service Request
*NIH F*	1 (2)	2 (5)	3 (2)	Consultee Service Request
*NIH RADx*	2 (4)	1 (3)	3 (2)	Consultee Service Request
*NIH other*	2 (4)	1 (3)	3 (2)	Consultee Service Request
*CFAR*	4 (8)	2 (5)	6 (4)	Consultee Service Request
*CDC*	2 (4)	1 (3)	3 (2)	Consultee Service Request
*CHRP/IS*	2 (4)	0 (0)	2 (1)	Consultee Service Request
*PCORI*	1 (2)	2 (5)	3 (2)	Consultee Service Request
*VA*	4 (8)	1 (3)	5 (4)	Consultee Service Request
*Internal/Institutional Funds*	3 (6)	0 (0)	3 (2)	Consultee Service Request
*Private/Foundation*	1 (2)	0 (0)	1 (1)	Consultee Service Request
*Other*	3 (6)	2 (5)	5 (4)	Consultee Service Request
*How valuable was consultation?*				
*"Very Valuable"*	28 (97)	13 (100)	41 (98)	Consultee Satisfaction Forms
*"Somewhat valuable"*	1 (3)	0 (0)	1 (2)	Consultee Satisfaction Forms
DISC Consultation Outcomes				
*Consultation Outcomes Forms*				
*# Consultations with Consultee form complete*	67	68	135	Consultee Satisfaction Forms
*# Consultations with Consultant complete*	68	68	136	Consultant Outcomes Forms
*# Consultations with both Consultee and Consultant complete*	67	68	135	
*# Consultations with no outcome forms*	0	2	2	
*Did DISC Consultation result in any of the following?*				
*"Grant Submission - completed"*	29 (43)	31 (46)	60 (44)	Consultant Outcomes Forms
*"Grant Submission - in progress"*	4 (6)	7 (10)	11 (8)	Consultant Outcomes Forms
*"Developed new scientific collaborator(s)"*	1 (1)	7 (10)	8 (6)	Consultant Outcomes Forms
*"Paper Submission - in progress"*	1 (1)	3 (4)	4 (3)	Consultant Outcomes Forms
*"Paper Submission - completed"*	1 (1)	4 (6)	5 (4)	Consultant Outcomes Forms
*“Formed community partnership”*	0 (0)	0 (0)	0 (0)	Consultant Outcomes Forms
*“Operationalized new program or refined existing program”*	3 (4)	4 (6)	7 (5)	Consultant Outcomes Forms
*“Scientific or Community Conference Presentation”*	1 (1)	5 (7)	6 (4)	Consultant Outcomes Forms
*“Other”*	8 (12)	11 (16)	19 (14)	Consultant Outcomes Forms
*What is the current status of the grant submission?*				
*“Funded”*	13 (19)	11 (16)	24 (18)	Consultant Outcomes Forms
*“Request to revise and resubmit”*	6 (9)	1 (1)	7 (5)	Consultant Outcomes Forms
*“Rejected”*	8 (12)	6 (9)	14 (10)	Consultant Outcomes Forms
*“Still pending or under review”*	4 (6)	13 (19)	17 (13)	Consultant Outcomes Forms
*“Other”*	0 (0)	6 (9)	6 (4)	Consultant Outcomes Forms
DIS Consultation-Scientific Outputs				
*DISC Consultation Timetracking*				
*Hours per Project*	9.79 (16.5)	6.39 (10.7)	8.13 (13.7)	Clockify Time Tracker
*Total Hours*	519	492	1011	Clockify Time Tracker
*DISC Member Consultation Satisfaction*				
*5= Strongly Agree, 1=Strongly Disagree*				
*The consultation meeting was scheduled in a timely manner.*	4.8 (0.8)	5 (0)	4.8 (0.7)	Consultee Satisfaction Forms
*My immediate needs were addressed during this first consultation.*	4.6 (1.0)	4.9 (0.3)	4.7 (0.8)	Consultee Satisfaction Forms
*The consultation meeting was useful.*	4.8 (0.8)	5 (0)	4.9 (0.7)	Consultee Satisfaction Forms
*The consultant addressed my questions clearly and completely.*	4.7 (0.9)	5 (0)	4.8 (0.7)	Consultee Satisfaction Forms
*I understand my next steps or action items.*	4.8 (0.8)	4.9 (0.3)	4.8 (0.7)	Consultee Satisfaction Forms
*There is a clear continuation plan to keep my work moving forward.*	4.6 (1.0)	4.9 (0.4)	4.8 (0.7)	Consultee Satisfaction Forms
*Resources or tools mentioned during the consultation were shared afterwards.*	4.7 (0.8)	5 (0)	4.8 (0.7)	Consultee Satisfaction Forms
*The consultant actively listened to my questions and description.*	4.8 (0.8)	5 (0)	4.9 (0.6)	Consultee Satisfaction Forms
*Connections and/or collaborations offered during the meeting were shared afterwards.*	4.7 (0.9)	4.8 (0.6)	4.7 (0.8)	Consultee Satisfaction Forms
*I would recommend the DISC consultation service to my colleagues.*	4.8 (0.8)	5 (0)	4.9 (0.7)	Consultee Satisfaction Forms

DISC = UC San Diego Dissemination and Implementation Science Center; DIS = dissemination and implementation science; NIH = national institutes of health; R, P, UG, F, RADx = types of grants funded by the national institutes of health; CFAR = center for aids research; CDC = centers for disease control; CHRP -; IS - ; PCORI = patient-centered outcomes research institute; VA = veterans affairs.

1 The denominators are as follows: “Consultation types” is out of the number of completed consultations for that year. Types of grants is out of the number of consultations for grant support. “How valuable consultation were” is out of the number of respondents to the question (*n* = 29). “Consultation results” and “current status of grant submission” are also out of number of completed consultations for that year.

### DISC Member Feedback

Table 4 includes qualitative responses to the DISC Annual Evaluation surveys and the DISC Workshop Evaluation forms. Common themes across respondents included the desire for increased diversity, student-focused content and opportunities, increased collaboration opportunities, online resources, methods-focused workshops, interest in practical application of DIS, need for assistance with DIS grant writing, interest in equity-oriented research and practice, and interest in advancing DIS skills to move from a novice to more advanced DIS user.

**Table 4.** Open qualitative feedback from disc annual evaluation and disc workshop evaluation

	DISC evaluation year 1	DISC evaluation year 2	DISC workshop evaluation form
Themes	Example responses	Example responses	Example responses
Student-focused content and opportunities	*"I really love the journal club!! Also, Involving students and young professionals, including connection with local programs"*	*More graduate student focused guidance and material!* *"Free workshops, trainings, and presentations! I'm a grad student with limited professional development funds."*	None
Desire for increased diversity	*More discussions from scholars of color and scholars from underrepresented backgrounds*	*Presentations by BIPOC scholars in the field*	*How to amplify the impact of community perspectives*
Increased community engagement and collaboration opportunities	*"Connection or open office hours of sorts to learn more about ways to engage and get connected"* *I would love a social hour from time to time to help build our community despite the pandemic.* *Project development work groups. Groups that come together to talk about different projects and what are the best methods to utilize for that project.*	*More ways to engage early career folks, such as training opportunities and mentorship.*	*Exploring how to forge strong partnerships with communities and with investigators across disciplines (to engage in transdisciplinary DIS work)*
More online supports and resources	*I'm just starting out with DISC, perhaps an "introductory" resource of some kind (e.g., reading list, suggested pages on website) might be helpful!*	*Asynchronous training resources with live support/input if needed*	*More practical applications of development of implementation strategies (and assessment)*
Desire for methods-focused workshops	*More short methods seminars at no cost* *More advanced workshops and trainings related to mixed methods, community academic partnerships, applying DIS frameworks and the formative evaluation process*	*Short trainings on specific DIS methods*	*Qualitative methods (e.g., conducting focus groups, interviews (coding methodology). Applying models and frameworks in developing new projects.*
Interest in practical application of DIS	*Short, practical trainings*	*More workshops with practical, hands on activitites. More Implementation Science Seminars.*	*Practical application of theories, frameworks and models*
Need for assistance with DIS grant writing	*Longitudinal grant workshopping for DIS-related grants.*	*Support in DIS grant proposals*	*Hybrid Model Research Design and Grant Writing*
Advancing DIS skills from beginner to more advanced	*More training in DIS models/ skills. I would love to participate in a lecture series that provides an intermediate introduction to new methods in the field.*	*I would love a clear road map to improve my knowledge and skills in implementation science. I have such limited time to engage in professional development activities, it would be really helpful if DISC could support me in figuring out what I know and what I do not, and then engage in targeted activities to develop my skills and knowledge in the weaker areas.*	*Resources for moving from beginner to intermediate DIS knowledge/activities*
Interest in equity-related research and practice within DIS	*More equity-focused work.*	*More presentations and workshops focused on evaluating equity related outcomes of policy, systems, organizational-level implementation science projects.*	*Continuing the focus on equity as central to DIS.*

DISC = UC San Diego Dissemination and Implementation Science Center; DIS = dissemination and implementation science.

## Discussion

Increased demand for and investment in DIS has led to an accelerated growth of programs focused on DIS training, mentorship, and capacity building [2,5]. To our knowledge, there has yet to be a published report outlining a process evaluation and results for a DIS capacity building program. The objectives of this process evaluation were to (1) assess how engagement in DISC activities translates into scientific products, outputs, and outcomes and (2) explore how to improve the DISC using feedback from members. To facilitate this evaluation, it was helpful to develop an organizing DLM to capture academic dissemination products and scientific outcomes resulting from or impacted by DISC activities. We mapped the measures and metrics of the process evaluation onto the domains of the DLM to identify gaps in evaluation plans.

Overall, engagement in the DISC was high with nearly all members participating in at least one activity intended to build capacity for DIS research and practice. Technical assistance offerings such as Journal Club, Implementation Science Seminars, and DISC Workshops were well received with high satisfaction ratings. Journal Club was most consistently offered with expanding participation from Year 1 to Year 2. Technical assistance activities may be most popular as there are likely few opportunities for discussion, collaboration, and practical application of DIS content outside of academically housed, DIS capacity building programs [8]. DISC participation also appears to be associated with diverse outputs, with grant submission most frequently endorsed. Fewer respondents endorsed outputs like publications and new scientific collaborations. This is not surprising because the largest cross-section of DISC members is academic faculty who are rewarded for or expected to seek funding; however, strengthening networking opportunities for members to develop new collaborations is a key area of focus to expand capacity and reach of DISC.

Compared with other DISC activities like seminars or workshops, the consultation service absorbed more personnel time and administrative resources. Although consultation has been free for members, it has become increasingly difficult to justify providing free consultation due to necessary time, resource, and personnel costs. Starting in 2022, the DISC team reorganized the consultation system and (1) requires a published (on our website) number of days or weeks (depending on the nature of the request) required for consultation requests, (2) notifies consultees that a fee (via institutional recharge) may be required beyond the first session, and (3) invites consultees to review the DISC website for information about the specifics of the consultation process. Information on the website also helps consultees prepare to use the intake most efficiently. Through our multimethod evaluation, we learned that members want shorter, methods-focused seminars and workshops targeting specific skills and practical application of DIS knowledge and frameworks. This might also help to alleviate burden from the consultation system. Actionable steps to build capacity within individuals might be to prioritize technical assistance and identify streamlined approaches to facilitate DIS grant writing through targeted writing workshops, “office hours” or Organized Writing Leagues [5]. These modifications are hypothesized to lead to greater academic productivity and successful grants resulting in expanded individual capacity to conduct DIS research and practice.

Strengths of the evaluation include multiple methods for evaluation consisting of member self-report, internal data tracking, and qualitative feedback. Long-term tracking that solely relied on consultee-report was challenging due to low response to outreach and follow-up surveys. Starting in 2023, we have now implemented an annual outreach to each consultee to schedule a 15-minute post-consult outcome call with specific questions for consultants to triangulate consultee self-report and maximize outcomes data. Regarding limitations, data from members were largely self-reported resulting in inconsistent missingness when respondents skipped questions. Also, missing data for race/ethnicity in Year 1 were greater than expected due to this question being added after the survey was distributed. We also did not include data from community partners in this first evaluation, but we do plan to collect data from community partners, advisors, regional and national collaborators in subsequent evaluations. We also included less qualitative data than we anticipated, due to challenges with repeated requests of our members and limited bandwidth.

**Figure 2.**
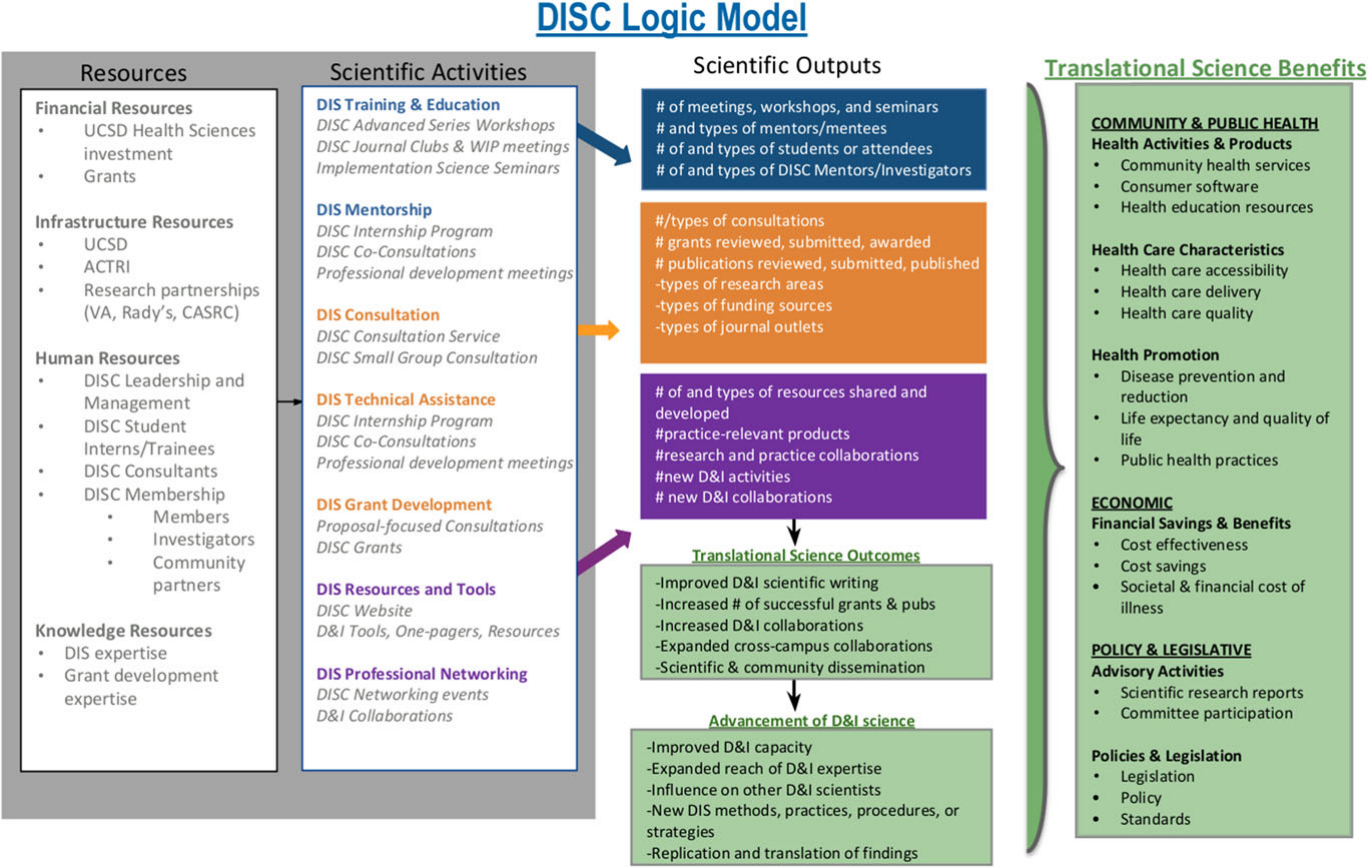
Prototype of future UC San Diego Dissemination and Implementation Science Center (DISC) logic model.

The ultimate goal of the DISC is to maximize DIS capacity among researchers and institutions in order to bring about broad and measurable population health impact. The Translational Science Benefits Model (TSBM) was developed to aid institution evaluations of translational outcomes including population health and dissemination outcomes like policy change [7]. This model has the potential to expand DIS program evaluation to beyond traditional academic outcomes to assess public health impacts within health services, health care delivery, public health practices, policy, and economic domains. In the future, we plan to offer activities to more intentionally lead to population-level translational science benefits (TSBs) like consultation for community partners and non-profits, seminars on strengthening public–private partnerships, resources targeting non-academic audiences, and workshops tailored to the burgeoning field of policy DIS. We also plan to incorporate TSBs into our current DLM and expand measurement of TSBs through consultation evaluations, annual membership surveys, DISC grantee reports, and DISC member news and events (see Fig. 2).

The DISC process evaluation has been an iterative, learning experience, and we are still trying to find the optimal balance of how much data we collect while not overburdening members, while continuing to stay apprised of member engagement and viewpoints and document impact. We hope for the evaluation to be pragmatic, actionable, feasible, and effective, which necessitates continuous discussion and consensus building to weigh pros and cons, optimize data collection, and maximize learnings.

### Recommendations for DIS Capacity Building Programs

Comprehensive and longitudinal evaluation of program activities and linking these activities to scientific outputs, community impacts, and longer term scientific and population health outcomes can be a useful way to explore how well DIS capacity building programs align with institutional and community priorities. We recommend that DIS programs include multiple data sources collected at varying frequencies to flexibly evaluate activities and inform participants from the outset that evaluation will be involved. Evaluation information is important and can guide refinement of activities and alignment of resources. We invite DIS programs to consider creating their own model or framework in collaboration with their institutional, clinical, and community partners. A logic model and linked evaluation can be instrumental in supporting communication with institutional decision makers and community partners and identifying gaps in activities and resources. Tracking DIS outcomes using a model or framework may also be effective in advocating for internal funding or infrastructural supports, leading to more sustained institutional-level capacity building.

## Conclusions

In summary, the DLM facilitated a comprehensive process evaluation of the DISC and helped us to understand how current activities may lead to expanded DIS capacity. Actionable steps to expand DIS capacity include prioritizing technical assistance, strengthening networking opportunities, identifying streamlined approaches to facilitate DIS grant writing through targeted writing workshops, as well as “office hours” or organized writing leagues, and building TSB activities and measurement.

## Supporting information

Viglione et al. supplementary material 1 Viglione et al. supplementary material

Viglione et al. supplementary material 2 Viglione et al. supplementary material

## Data Availability

The datasets used during the current study are available from the corresponding author upon request.
