# Supplementary material for: Process evaluation of an academic dissemination and implementation science capacity building program
Source: J Clin Transl Sci. 2023 Sep 15;7(1):e207. doi: 10.1017/cts.2023.630 (PMC10603357; doi:10.1017/cts.2023.630)
Supplement: Viglione et al. supplementary material 1 — Viglione et al. supplementary material [file S2059866123006301sup001.pdf]

DISC Annual Membership Evaluation Survey

Start of Block: DISC Consultation 6-Month Evaluation

Q90 <div>Please enter the last 4 digits of your cell phone number so we ensure that we receive only one response from each person.</div>

____

Q69 What is your current DISC membership level? <div>Note: You can check if you are a DISC Investigator on our website.</div>

○ Member

○ Investigator

○ Unsure

Q86 <p>What is your current level of experience with D&I research? <o:p></o:p></p>

○ I am a novice (I have not engaged in any activities related to D&I research)

○ I have advanced beginner D&I research skills (e.g., I have participated in some D&I training activities and may have contributed to a D&I proposal/project)

○ I have intermediate D&I research skills (e.g., I have engaged in D&I-related activities in the past but have not led a proposal/project with D&I research as its main focus)

○ I have advanced D&I research skills (e.g., I have led grant(s)/project(s) with D&I research as its main focus)

Q70 As part of your DISC Membership, which activities and services have you participated in or completed over the past 12 months? Please select all that apply.

- ☐ Participated in Monthly DISC Journal Club and Works in Progress
- ☐ Utilized resources on the DISC website to support my work in research and/or practice
- ☐ Received consultation about D&I grant proposals, manuscripts, and/or projects
- ☐ Utilized D&I tools or opportunities shared in the DISC Monthly Newsletter
- ☐ Attended Annual Advanced D&I Methods Workshop
- ☐ Attended external D&I training or educational events publicized by the UC San Diego DISC
- ☐ Presented at a DISC event (Annual Proposal Bootcamp, Annual Advanced D&I Methods Workshops, Implementation Science Seminar Series, D&I Journal Club and Works in Progress)
- ☐ Connected with new potential collaborator in D&I Science
- ☐ Engaged with the UC San Diego DISC on Twitter (e.g., followed, liked, re-tweeted, etc.)
- ☐ Provided a guest lecture in the annual Dissemination and Implementation Science in Health (DISH, FPM 291) course
- ☐ Served as an ad-hoc consultant for the DISC Consultation Service
- ☐ Submitted a DISC D&I Pilot Grant Proposal
- ☐ Applied for DISC Conference, Training or Publication Award
- ☐ Reviewed DISC Conference, Training or Publication Awards

☐ Reviewed DISC D&I Pilot Grant Proposals

☐ Served as key personnel on a grant proposal that is submitted through the UC San Diego DISC

☐ Other ____

Q12 Were you supported with any of the following activities through your DISC membership?

- ☐ Received formal mentoring for career award
- ☐ Grant submission - completed
- ☐ Grant submission - in progress
- ☐ Funded grant
- ☐ Paper submission - in progress
- ☐ Paper submission - completed
- ☐ Published paper
- ☐ Developed new scientific collaborator(s)
- ☐ Formed community partnership(s)
- ☐ Operationalized new program or refined existing program
- ☐ Scientific or Community Conference Presentation—in progress
- ☐ Scientific or Community Conference Presentation—completed
- ☐ Other ____

Q24 Where was (or will be) the grant submitted?

- ☐ National Institutes of Health
- ☐ Centers for Disease Control
- ☐ Veterans Affairs
- ☐ Health Resources and Services Administration
- ☐ Agency for Healthcare Research and Quality
- ☐ Patient-Centered Outcomes Research Institute
- ☐ William T. Grant Foundation
- ☐ Robert Wood Johnson Foundation
- ☐ State/local government agency
- ☐ Other private foundation
- ☐ Internal academic institution
- ☐ Other: ____

Q23 If yes (grant submission): What is the status?

- ◯ Funded
- ◯ Positively evaluated but not funded (revise and resubmit)
- ◯ Rejected
- ◯ Still pending or under review
- ◯ Other ____

Q25 To which journal was your publication submitted and/or accepted?

____

Q14 If YES (publication submission): What is the status?

- ◯ Accepted
- ◯ Positively evaluated but not published (revise and resubmit)
- ◯ Rejected
- ◯ Still pending or under review
- ◯ Other ____

Q87 Did you participate and/or present at the Implementation Science Seminar over the past 12 months?

- ☐ Presented
- ☐ Participated
- ☐ Did not attend

Q75 Did you participate and/or present at one of the DISC Journal Club and Works in Progress meetings over the past 12 months?

- ☐ Presented
- ☐ Participated
- ☐ Did not attend

Q68 How can we improve the DISC Journal Club and Works in Progress experience?

____

____

____

____

____

Q88 How can we improve the Implementation Science Seminar experience?

____

____

Q28 Have you used the DISC website (disc.ucsd.edu)?

- ◯ Yes
- ◯ No

Q16 Which sections of the UC San Diego DISC website have you used? (select all that apply)

- ☐ News & Events
- ☐ Tools & Resources
- ☐ Research Advancement
- ☐ Training & Education

Q18 Which of these D&I opportunities and resources would you find of value? (select all that apply)

- ☐ Online resources to support the consultation (i.e. interactive tools, e-books on content/models, etc.)
- ☐ Local conferences on D&I topics
- ☐ Formal graduate level course
- ☐ D&I certification program
- ☐ Access to online articles, websites, trainings
- ☐ DISC Consultation
- ☐ Other ____

Page Break

Q64 Please rate your agreement with the following statement: DISC is actively promoting equity through their various trainings and initiatives. <div> </div> <div>When we use the term <u>equity</u>, we mean the condition that would be achieved if one's identity (e.g., race, age, income, gender) no longer predicted, in a statistical sense, how one fares. We are thinking about equity as one part of justice, and thus we also include work to address root causes of inequities, not just their manifestation. This includes elimination of policies, practices, attitudes, and cultural messages that reinforce differential outcomes by identity or that fail to eliminate them. - Source: Center for Assessment and Policy Development</div> <div> <div>To learn more about how the DISC intends to address equity, please read this statement.</div> </div>

- ○ Strongly agree
- ○ Somewhat agree
- ○ Neutral (neither agree nor disagree)
- ○ Somewhat disagree
- ○ Strongly disagree

Q71 The DISC Community has identified 3 health equity priorities for 2021. Please order these priorities in terms of importance from 1 (most important) to 3 (least important). Click and drag to re-order.

______ Priority A: DISC- hosted panel discussions with community voices present on improving racial equity using D&I

______ Priority B: Community-based D&I lab aimed at supporting community-led programs and projects (rather than researcher-initiated projects)

______ Priority C: DISC outreach presentations on "Intro to D&I" and how community members can take advantage of DISC offerings and trainings

---

Q89 What other actions/initiatives should the DISC take to address health equity?

____

____

____

____

____

Q65 In the coming year, we are hoping to broaden our outreach to organizations and agencies embedded within communities located in San Diego and Imperial Counties. Do you currently work with community-based organizations, non-profit organizations, public agencies, etc. that might benefit from UC San Diego DISC services or trainings?

- ◯ Yes
- ◯ No

Q66 If yes, what organization(s) specifically?

- ◯ Organization Name: ____
- ◯ Organization Name: ____
- ◯ Organization Name: ____

Q76 Would you be interested in connecting a member of the DISC Team with these organization(s) to share our DISC services or conduct a short DISC presentation?

- ◯ Yes
- ◯ Maybe
- ◯ No

Q84 If you are comfortable, please enter your e-mail address and we will e-mail you to facilitate an introduction with the listed organization(s).

____

Q79 Over the past 12 months, have you turned to someone for information and guidance on D&I research and practice?

○ Yes

○ No

Q78 <div>Over the past 12 months, who have you turned to for advice for information and guidance on D&I research and practice? Please add up to three names and their affiliations. </div><div>
</div><div>Note: We will not be publishing names or contacting those listed. We are using this information to generate an advice-seeking network map for D&I from the perspective of our DISC Members and Investigators. </div>

○ Full Name 1: ____

○ Institution or Affiliation 1: ____

○ Full Name 2: ____

○ Institution or Affiliation 2: ____

○ Full Name 3: ____

○ Institution or Affiliation 3: ____

Q44 How likely are you to recommend engaging with DISC services and opportunities to someone in your professional network?

- Extremely likely
- Somewhat likely
- Neutral (Neither likely nor unlikely)
- Somewhat unlikely
- Extremely unlikely

Q27 What could improve your experience with the DISC?

____

____

____

____

____

Q77 What would you like to see more of from the DISC in 2021?

____

____

____

____

____

Page Break

Q81 If you would like to be entered into the raffle for a $25 gift card of your choice (Amazon or Starbucks), please enter your name and e-mail address.

- ○ Last Name ____
- ○ First Name ____
- ○ Email Address ____

Q37 Title (select all that apply)

- ☐ Professor
- ☐ Associate Professor
- ☐ Assistant Professor
- ☐ Postdoctoral Trainee
- ☐ Graduate Student
- ☐ Undergraduate Student
- ☐ Clinician/health care provider
- ☐ Non-faculty researcher
- ☐ Research staff (Project Coordinator or Manager)
- ☐ Project Scientist
- ☐ Research Scientist
- ☐ Other (specify): ____

Department (select all that apply)

- ☐ Affiliated Centers (e.g. Center of Excellence for Stress & Mental Health, SDSU ARPE, etc.)
- ☐ Anthropology
- ☐ Engineering
- ☐ Nursing
- ☐ Medicine (Includes all sub-specialties)
- ☐ Pathology
- ☐ Pediatrics
- ☐ Pharmacy
- ☐ Psychiatry
- ☐ Psychology
- ☐ Public Health
- ☐ Obstetrics and Gynecology
- ☐ Ophthalmology
- ☐ Social Work
- ☐ Surgery
- ☐ Other ____

DISC Affiliated Centers (select all that apply)

- [ ] Center of Excellence for Stress & Mental Health
- [ ] Child and Adolescent Services Research Center
- [ ] San Diego Center for Aids Research
- [ ] San Diego State University - Administration, Rehabilitation, and Postsecondary Education
- [ ] San Diego State University - Interwork Institute
- [ ] Chadwick Center
- [ ] San Diego State University - Institute for Behavioral and Community Health
- [ ] Qualcomm Institute
- [ ] Other ____

Medicine (Please select sub-specialty area(s))

- [ ] Allergy and Immunology
- [ ] Cardiology
- [ ] Dermatology
- [ ] Endocrinology
- [ ] Family Medicine
- [ ] Gastroenterology
- [ ] General Internal Medicine
- [ ] Geriatrics
- [ ] Infectious Disease
- [ ] Neurology
- [ ] Oncology
- [ ] Obstetrics & Gynecology
- [ ] Pulmonary and Critical Care
- [ ] Rheumatology
- [ ] Other ____

Q72 <p>Diversity is a source of strength for the UC San Diego DISC. Our diverse backgrounds enhance our ability to achieve our core mission. </p><p>
</p> Please select which groups you identify with (select all that apply):

- American Indian/Alaskan Native
- Asian (Not Pacific Islander)
- Black/African American (Not Hispanic)
- Caucasian/White (Not Hispanic)
- Filipino/Pilipino
- Latin American/Latino/Latinx
- Mexican/Mexican American/Chicano
- Native Hawaiian/Other Pacific Islander
- Other Spanish/Spanish American
- Prefer to self describe ____
- Prefer not to answer

Q74 Please indicate your current gender identity (please select one):

- ○ Male
- ○ Female
- ○ Non-binary / Genderqueer
- ○ Prefer to self describe ____
- ○ Prefer not to answer

Q45 Anything else that you would like the DISC team to know?

____

____

____

____

____

End of Block: DISC Consultation 6-Month Evaluation
