# Supplementary material for: Process evaluation of an academic dissemination and implementation science capacity building program
Source: J Clin Transl Sci. 2023 Sep 15;7(1):e207. doi: 10.1017/cts.2023.630 (PMC10603357; doi:10.1017/cts.2023.630)
Supplement: Viglione et al. supplementary material 2 — Viglione et al. supplementary material [file S2059866123006301sup002.pdf]

DISC Logic Model Constructs

The final DISC Logic Model (DLM) highlights resources aligning most closely with the WUNDIR model (e.g., grants, types of center members, DIS expertise, etc.). Financial Resources include grants, investments, and internal institutional funding (e.g., from UC San Diego health sciences). Infrastructure resources include UC San Diego, ACTRI, and research partnerships like Veterans Affairs, Rady Children's Hospital, and the Child and Adolescent Services Research Center. Human Resources were subdivided into DISC Executive Leadership and Management, DISC student interns/trainers, DISC consultations, and the different DISC Member groups. Lastly, Knowledge was subdivided into specific DIS areas of expertise such as grant development.

The DLM enumerates scientific activities based on the WUNDIR activity categories with DIS training and education, DIS mentorship, DIS consultation, DIS technical Assistance, DIS grant development, DIS resources and tools, and DIS professional networking. Proximal and intermediate outcomes are from WUNDIR (e.g., improved DIS scientific writing, increased DIS collaborations, etc.). In each of the seven scientific activities, there are several examples such as DISC individual and small group consultation, DISC networking events, DISC Journal Club, and DISC Advanced Methods Workshops.

Scientific outputs include number of meetings, workshops, seminars, types of consultations, number of grants reviews, submitted, and awarded. This section helps to evaluate the more specific aspects of capacity building.
